# Successful Exogenous Expression of ATP8, a Mitochondrial Encoded Protein, From the Nucleus In Vivo.

**DOI:** 10.1093/geroni/igab046.2577

**Published:** 2021-12-17

**Authors:** David Begelman, Bhavna Dixit, Caitlin Lewis, Mark Watson, Martin Brand, Amutha Boominathan

**Affiliations:** 1 SENS Research Foundation Research Center, Novato, California, United States; 2 SENS Research Foundation Research Center, Mountain View, California, United States; 3 Buck Institute for Research on Aging, Novato, California, United States

## Abstract

Replicative errors, inefficient repair, and proximity to reactive oxygen species production sites make the mitochondrial DNA (mtDNA) susceptible to damage with time. mtDNA mutations accumulate with age and accompany a progressive decline in organelle function. We lack molecular biology tools to manipulate mtDNA, thus we explore the possibility in vivo of utilizing allotopic expression, or the re-engineering mitochondrial genes and expressing them from the nucleus, as an approach to rescue defects arising from mtDNA mutations. This study uses a mouse model with a mutation in the mitochondrial ATP8 gene that encodes a protein subunit of the ATP synthase. We generated a transgenic mouse with an epitope-tagged recoded and mitochondrial-targeted ATP8 gene expressed from the nucleus. Our results show that the allotopically expressed ATP8 protein in the transgenic mice is robustly expressed across all tested tissues, successfully transported into the mitochondria, and incorporated into ATP synthase. We are currently evaluating if allotopic expression of ATP8 will functionally rescue the behavioral and bioenergetic defects in ATP8 mutant mice. Translating allotopic expression technology into a mammal and demonstrating systemic functional rescue will lend credence to utilizing allotopic expression as a gene therapy in humans to repair physiological consequences of mtDNA defects that may accumulate with age.

